# Survival stratification in childhood rhabdomyosarcoma of the extremities: a derivation and validation study

**DOI:** 10.1038/s41598-020-62656-x

**Published:** 2020-03-30

**Authors:** Linchao Zhu, Ying Sun, Xuhui Wang, Lin Wang, Shufeng Zhang, Qinglei Meng, Xiaohui Wang

**Affiliations:** 1 Department of Pediatric Surgery, Henan Provincial People’s Hospital, Zhengzhou, 450000 Henan Province China; 2 Department of Clinical Laboratory, Third People’s Hospital of Henan Province, Zhengzhou, 450000 Henan Province China

**Keywords:** Cancer, Cancer therapy, Oncology

## Abstract

The objective of this study was to estimate overall survival in children with extremity rhabdomyosarcoma (RMS). In addition, we attempted to construct a nomogram to predict the prognosis in such patients using a population-based cohort. The national Surveillance, Epidemiology, and End Results (SEER) registry was used to identify a cohort of childhood RMS patients. A total of 197 patients with RMS were ultimately included. Multivariable analysis identified age group, N classification, M classification, and treatment combinations as independent predictive factors for patient overall survival. Candidate variables such as age group, N classification, M classification, and treatment combinations were used to fit the model. For overall survival, the bootstrap-adjusted c-index was 0.76 (95% CI, 0.73–0.80) for the nomogram. Furthermore, we performed recursive partitioning analysis for risk stratification according to overall survival, and 3 prognostic subgroups were generated (low, intermediate and high risk). Finally, we evaluated multimodal treatment based on the risk stratification according to the nomogram and IRSG prognostic stratification model. With regard to the entire cohort, overall survival in patients who received surgery and radiation was superior to that in patients who received surgery or radiation (p = 0.001). Regarding RPA and IRSG prognostic stratification, we found that the differences remained significant (p < 0.05) in patients with low-intermediate risk. However, the difference disappeared in patients with high risk (p > 0.05). We performed a population-based analysis of data from the SEER registry in an effort to identify prognostic factors and develop a nomogram in children with extremity RMS. The nomogram appears to be suitable for the survival stratification of children with RMS and will help clinicians identify patients who may be at a reduced probability of survival and assist them in making treatment and surveillance decisions. More studies concerning overall survival in children with RMS are needed to confirm and update our findings.

## Introduction

Childhood rhabdomyosarcoma (RMS), a heterogeneous group of soft tissue malignant tumors of mesenchymal cell origin, is the most common soft tissue sarcoma (STS) in children, with an annual incidence of 4.6 per million in those younger than 20 years of age^1^. It accounts for approximately 3.5% of malignancies among children aged 0 to 14 years and 2% of malignancies among adolescents aged 15 to 19 years^2^. Over the past 25 years, only 4292 eligible patients in 5 successive completed clinical protocols were recorded^3–7^. Thus, it is difficult to study childhood RMS, even though it is the most common form of STS in children.

Survival in patients with RMS is influenced by several factors, such as age at diagnosis, histology subtype, tumor size, tumor location, regional lymph node involvement, distant metastases, surgery and adjuvant therapy^7^. The first four generations of the Intergroup Rhabdomyosarcoma Study Group (IRSG) therapeutic trials (IRS I-IV) were developed by a new International Classification of Rhabdomyosarcoma (ICR) Committee (later the (IRSG)) that stratified patients into various treatment protocols, which greatly improved the overall survival^7^. As reported, the overall 3-year failure-free survival and overall survival rates in children with nonmetastatic RMS were 77% and 86%, respectively^8^. This improvement in survival is largely due to the improvement in treatments^8,9^ and the implementation of comprehensive, optimal treatment strategies based on prognostic stratification.

Due to a lack of data, to date, optimal prognostic stratification for childhood RMS of the extremities remains far from being established. Studies in which nomograms were constructed and validated to predict prognosis in such patients have not been reported. Moreover, the clinical grouping and staging systems of the IRSG were established in the last century and need to be reevaluated^7^.

In this study, we performed a population-based analysis of data from the Surveillance, Epidemiology, and End Results (SEER) registry to identify prognostic factors and to develop a prognostic stratification model in children with extremity RMS. Furthermore, multimodal treatment was evaluated based on the prognostic stratification model.

## Materials and Methods

### Data source and case selection

The national SEER registry has been described in detail elsewhere. The SEER program actively collects information on patient demographics, tumor diagnosis, and treatment-related risk factors from 18 registries, covering approximately 27.8% of the U.S. population (based on the 2010 census)^10^. Children (aged 0–19 years) diagnosed with RMS (histologically proven) between 1973 and 2014 were eligible for this study. The diagnosis was based on the primary tumor site using the third edition of the International Classification of Diseases for Oncology (ICDO-3). For this study, we included patients with codes 8900, 8901, 8902, 8910, 8912, 8920 and 8921. Additionally, we excluded patients who did not receive chemotherapy. The collected data in this study included age, sex, race, tumor location, TNM classification, tumor size, grade, histology, surgery, radiation, treatment combinations, the IRSG staging system, the IRSG surgical-pathologic grouping system and the IRSG risk stratification model. To evaluate the therapeutic significance of the surgery, we also excluded patients with unknown surgery information. This study was deemed exempt from review by the Institutional Review Boards of Third People’s Hospital of Henan Province.

### Statistical analysis

Categorical variables are expressed as n (%), and continuous variables are presented as the means ± standard errors (means ± SEs). Survival was estimated with the Kaplan-Meier method. The survival analysis was performed with log-rank tests and Cox proportional hazards regression models for univariable and multivariable analyses, respectively. Clinically relevant variables with p values < 0.2 in univariable analysis and those that could be related to the outcome were considered for inclusion in the final Cox proportional hazards multivariable regression model. In the Cox proportional hazards multivariable regression model, we verified the proportional hazards assumption.

A nomogram was formulated based on the results of the Cox proportional hazards regression model. The performance of the nomogram was measured by its discriminatory ability and calibration by the bootstrapping method with 1,000 resamples. Comparisons between the nomogram and other staging systems were estimated by the bootstrap-adjusted c-index to obtain an unbiased measure of the ability of the nomogram and IRSG prognostic stratification model to discriminate between patients. The larger the c-index value was, the more accurate the prognostic stratification. Calibration was performed to examine how well the model-based predicted probability of survival agreed with the observed probabilities. Furthermore, a recursive partitioning analysis (RPA) method was adopted to stratify the patients according to the probability of overall survival. Finally, multimodal treatment was evaluated based on risk stratification according to the nomogram and IRSG prognostic stratification model.

All statistical analyses were performed using R version 3.4 (http://www.r-project.org/), and the main packages used for data analysis in this study were rms (nomogram) and Hmisc (for comparisons between the nomogram and other prognostic systems). Two-sided p-values less than 0.05 were considered statistically significant.

## Results

A total of 197 patients with RMS were included in the study. The overall patient demographics, tumor characteristics and treatment details are summarized in Table 1. There were 109 males and 88 females, with a mean age of 8.34 ± 5.601 years. The mean survival time was 63.48 ± 52.090 months during the follow-up period. The 2-, 3-, and 5-year overall survival rates were 77.1% (95% CI, 0.74–0.80), 67.9% (95% CI, 0.64–0.71), and 55.7% (95% CI, 0.52–0.60), respectively.

**Table 1:** Descriptive statistics and univariate analysis for overall survival in the childhood rhabdomyosarcoma cohort.

Characteristic (n = 197)		Value, mean ± SE or n (%)	HR	95% CI	p
Age (years)		8.34 ± 5.601	1.068	1.028–1.110	0.001*
Age group	≥10	88 (44.7)	baseline		
	1–9	83 (42.1)	0.610	0.392–0.952	0.029*
	≤1	26 (13.2)	0.347	0.148–0.810	0.014*
Gender	Male	109 (55.3)	baseline		
	Female	88 (44.7)	1.018	0.667–1.554	0.934
Race	White	143 (72.6)	baseline		
	Black	36 (18.3)	1.176	0.686–2.016	0.556
	Other	18 (9.1)	1.443	0.739–2.820	0.283
Year of diagnosis	2007–2014	98 (49.7)	baseline		
	1998–2006	99 (50.3)	0.887	0.577–1.362	0.583
Tumor location	Upper limb, shoulder	90 (45.7)	baseline		
	Lower limb, hip	107 (54.3)	0.932	0.611–1.421	0.744
Tumor size	≤5 cm	82 (41.6)	baseline		
	>5 cm	102 (51.8)	2.174	1.364–3.465	0.001*
	Unknown	13 (6.6)	2.949	1.271–6.482	0.012*
N classification	All negative	90 (45.7)	baseline		
	Positive	65 (33.0)	2.702	1.637–4.458	<0.001*
	Unknown	42 (21.3)	2.393	1.379–4.152	0.002*
M classification	M0	130 (66.0)	baseline		
	M1	63 (32.0)	5.019	3.252–7.747	<0.001*
	Unknown	4 (2.0)	0.656	0.090–4.777	0.678
Grade	Grade I/II/III	25 (12.7)	baseline		
	Grade IV	31 (15.7)	1.006	0.452–2.240	0.989
	Unknown	141 (71.6)	1.113	0.570–2.173	0.753
Histology	Embryonal	27 (13.7)	baseline		
	Alveolar	138 (70.1)	1.594	0.794–3.200	0.190
	Others	32 (16.2)	1.328	0.559–3.155	0.520
Surgery	surgery	136 (69)	baseline		
	No surgery	61 (31)	2.037	1.319–3.148	0.001*
Radiation	None/Unknown	42 (21.3)	baseline		
	Yes	155 (78.7)	0.781	0.478–1.277	0.324
Treatment combinations	Surgery without radiation	42 (21.3)	baseline		
	Surgery + radiation	94 (47.7)	0.547	0.316–0.947	0.031*
	Radiation without surgery	61 (31.0)	1.355	0.784–2.344	0.277
IRSG staging system^#^	Stage 2	55 (27.9)	baseline		
	Stage 3	69 (35.0)	2.784	1.382–5.608	0.004*
	Stage 4	69 (35.0)	8.453	4.328–16.512	<0.001*
	Unknown	4 (2.0)	1.199	0.155–9.292	0.862
IRSG grouping system	Group I/II	58 (29.4)	baseline		
	Group III/IV	137 (69.5)	1.772	1.074–2.924	0.025*
	Unknown	2 (1.0)	1.365	0.183–10.176	0.761
IRSG prognostic stratification	Low	22 (11.2)	baseline		
	Intermediate	119 (60.4)	0.615	0.297–1.276	0.192
	High	52 (26.4)	3.668	1.769–7.605	<0.001*
	Unknown	4 (2.0)	0.404	0.051–3.190	0.390
Survival months (month)		63.48 ± 52.090	NA	NA	NA

^#^By definition, an extremity RMS cannot be stage 1 (Supplementary Table 1).

*Statistical significance.

The new survival stratification model was developed from a median 46.0 months of follow-up in the SEER-based cohort. As shown in Table 1, in the univariable analysis, we identified 9 factors that were associated with overall survival. Furthermore, we included age group, tumor size, N classification, M classification, histology, treatment combinations and variables with potential clinical significance (tumor location, grade) in the final Cox proportional hazards multivariable regression model. Surgery and radiation were excluded because they were incorporated into the existing variable treatment combinations. We also did not include the IRSG staging system, IRSG surgical-pathologic grouping system or IRSG prognostic stratification model in the final Cox proportional hazards multivariable regression model because they are systematic prognostic systems containing several prognostic factors (Supplementary Tables 1 and 2). Furthermore, multivariable analysis identified age group, N classification, M classification, and treatment combinations as independent factors predictive of patient overall survival. Thus, age group, N classification, M classification, and treatment combinations were used to fit the model. For overall survival, the bootstrap-adjusted c-index was 0.76 (95% CI, 0.73–0.80) for the nomogram.

**Figure 1:**
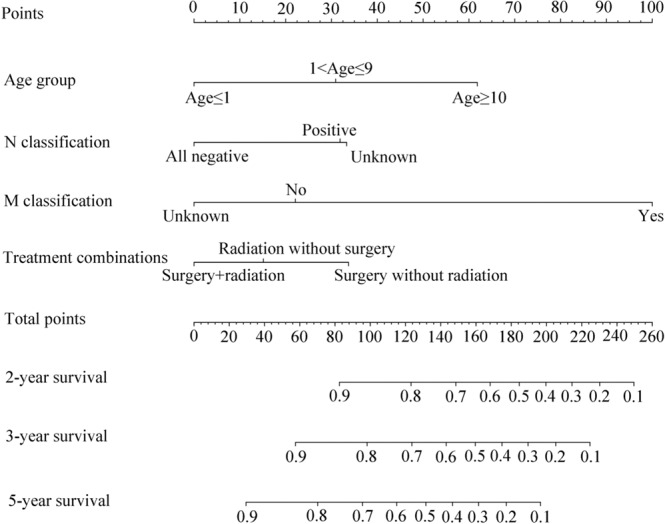
Nomogram for predicting 2-, 3- and 5-year overall survival. The instructions are as follows: locate a patient’s characteristics on the corresponding axis to determine how many points the patient receives. Sum the points achieved for each of the characteristics and locate this sum on total points axis. Draw a line straight down to identify the patient’s probability for 2-year survival, 3-year survival, and 5-year survival.

**Figure 2:**
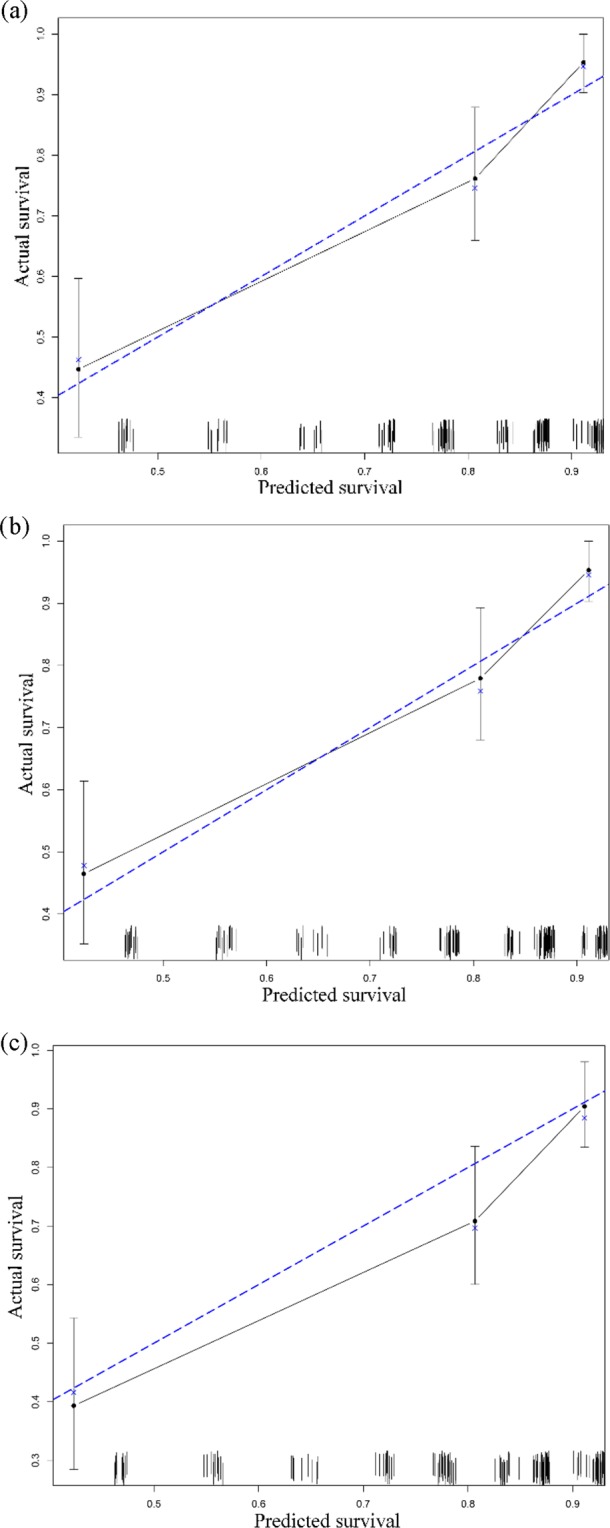
Calibration plot. (**a**) 2-year overall survival; (**b**) 3-year overall survival; (**c**) 5-year overall survival.

**Figure 3:**
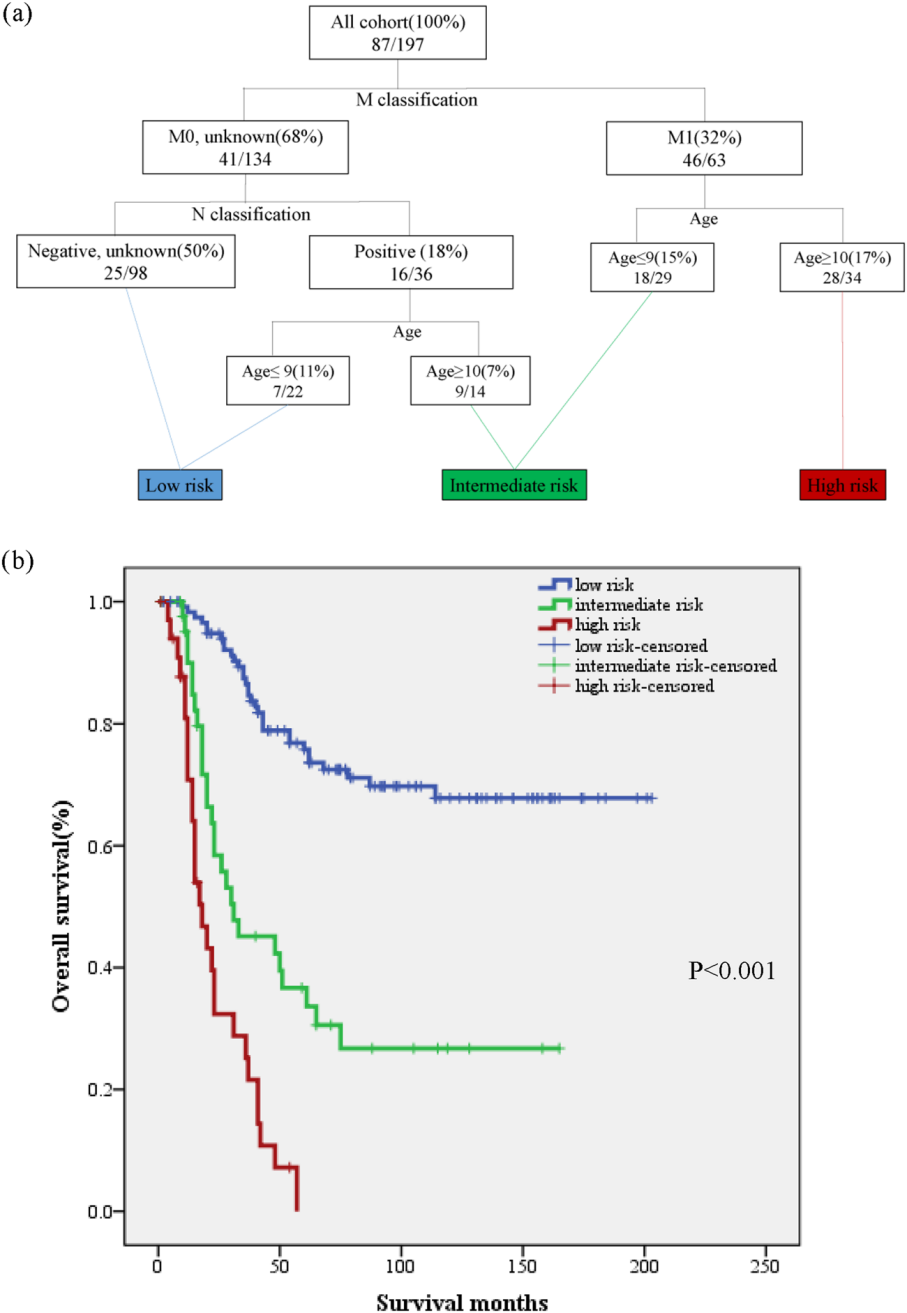
(**a**) Recursive partitioning analysis grouping into three risk stratifications for the prediction of overall survival. The ratios indicate the number of deaths divided by the number of patients at risk. (**b**) Kaplan–Meier curve for survival probability by recursive partitioning analysis risk group.

The weights and points associated with the nomogram are shown in Fig. 1. The calibration plots demonstrated good concordance between the predicted and actual survival at 2, 3, and 5 years (Fig. 2). Furthermore, we performed RPA for risk stratification according to overall survival. All independent risk factors were evaluated as potential split points. Ultimately, the tree was pruned to generate 3 prognostic subgroups using the endpoint of overall survival. As depicted in Fig. 3a, we identified splits corresponding to M classification, N classification and age group. Thus, we partitioned the patient population into three risk strata – low risk, intermediate risk and high risk – according to overall survival. In the entire cohort, the 5-year overall survival rates of patients with low risk, intermediate risk, and high risk were 75.8% (95% CI, 0.72–0.80), 36.7% (95% CI, 0.29–0.45), and 0%, respectively. As depicted in Fig. 3b, the RPA risk system demonstrated statistically significant difference in terms of survival probability (p < 0.001).

**Figure 4:**
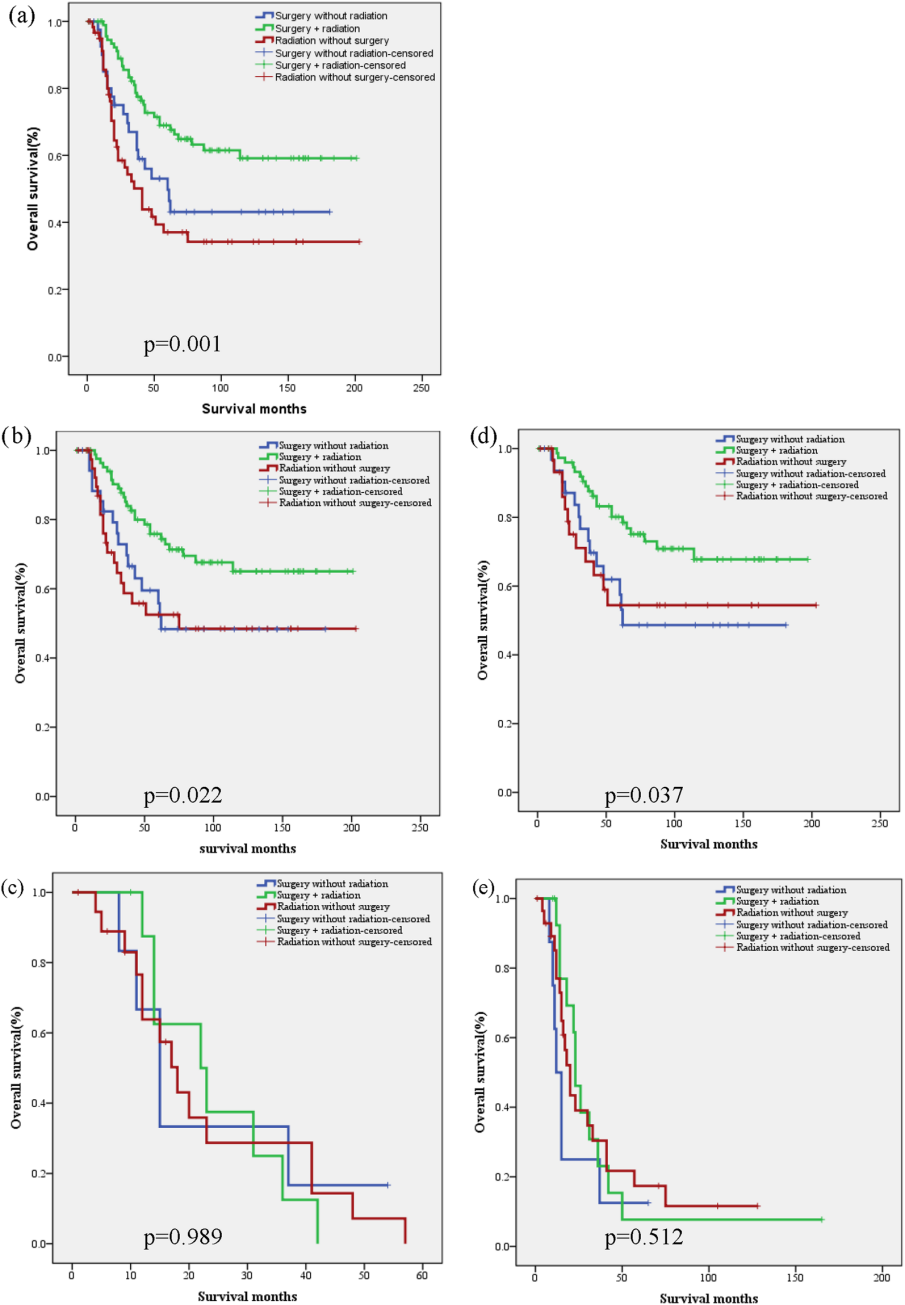
Multimodal treatment based on risk stratification. (**a**) The entire cohort; (**b**) low-intermediate risk (RPA stratification); (**c**) high risk (RPA stratification); (**d**) low-intermediate risk (IRSG prognostic stratification); (**e**) high risk (IRSG prognostic stratification).

Finally, we evaluated multimodal treatment based on the risk stratification according to the nomogram and IRSG prognostic stratification model (the IRSG staging system and IRSG surgical-pathologic grouping system were excluded because they were incorporated in variable “IRSG prognostic stratification” (Supplementary Table 2)). With regard to the entire cohort, overall survival in patients who received surgery and radiation was superior to that in patients who received surgery or radiation (p = 0.001, Fig. 4a). Regarding RPA stratification, we found that the differences remained significant (p = 0.022, Fig. 4b) in patients with low-intermediate risk. However, the difference disappeared in patients with high risk (p = 0.989, Fig. 4c). Similarly, regarding IRSG prognostic stratification, we found that the differences remained significant (p = 0.037, Fig. 4d) in patients with low-intermediate risk. However, the difference disappeared in patients with high risk (p = 0.512, Fig. 4e).

## Discussion

Childhood RMS has long been difficult to study due to both its rarity and its heterogeneity. The IRSG surgical-pathological grouping system and the IRSG staging system are based on 5 successive completed clinical protocols, and the use of these protocols has resulted in significant advantages for clinicians in the assessment and treatment of the disease. In this analysis, we report a population-based analysis assessing our newly built prognostic model in children with extremity RMS.

The results of the Cox model identified age group, N classification, M classification and treatment combination as important predictors of childhood RMS survival. These findings were expressed consistently in the nomogram and were then further evaluated. The calibration plots demonstrated good concordance between the predicted and actual survival at 2, 3, and 5 years (Fig. 2), and the bootstrap-adjusted c-index of 0.76 (95% CI, 0.73–0.80) suggested that the nomogram had a good capacity to discriminate among patients.

Studies have tried to explore the associations of age with patient survival and have shown shorter median survival^11^, lower long-term survival probabilities and worse overall/cause-specific/failure-free/event-free survival^8,9,12–15^ in older patients. Furthermore, Archer^16^ concluded that young children tend to have an increased incidence of second malignant neoplasms (SMNs), potentially as a result of a lower chance of death from RMS before developing SMNs and a longer survival time in which to develop SMN. However, Stevens *et al*.^9^ demonstrated that age had no impact on overall survival (p = 0.11), despite it having a significant impact on event-free survival (p = 0.04). However, a major limitation exists in this study because patients aged < 10 years accounted for 81% of the patient population. Previous studies have reached a consensus that age at diagnosis is an important prognostic factor for survival in childhood RMS, which is consistent with our conclusions. Part of the reason for the worse survival in older patients is that they tend to have less favorable risk situations, such as more aggressive alveolar histology, unfavorable tumor sites and more advanced clinical stage^14,17^. Studies have shown that pediatric RMSs have better sensitivity to chemotherapy than those diagnosed in adults. Even patients with more aggressive alveolar histology can achieve better survival with intensive chemotherapy^18^. As reported, complete remission was achieved in 93% of pediatric RMSs treated with chemotherapy^9^; In contrast, adult MRS patients have worse outcomes and are more treatment refractory^19^.

The IRSG surgical-pathological grouping system, IRSG staging system and IRSG prognostic stratification model are all primarily based on age at diagnosis, tumor size, lymph node involvement and distant metastasis. In this study, we created a nomogram for prognostic stratification. Prognostic stratification is important for informing treatment decisions, and the treatment strategy also greatly impacts patient survival^20,21^. Studies have shown that microscopic residual tumors invariably remain or disseminate in the majority of patients despite a total gross resection^11,22^. Current treatment recommendations for patients with extremity sarcomas are an initial complete resection to achieve negative margins if feasible without compromising form and function^15^. Furthermore, it is widely accepted that the treatment strategy should include multiagent chemotherapy and radiotherapy in addition to surgery to achieve significantly better effects. As described in Table 2, we concluded that patients who underwent surgery alone had worse survival than patients who underwent surgery combined with radiotherapy (p = 0.017). A study by Eduardo *et al*. reached a similar conclusion^11^. However, a study by La *et al*.^15^ showed that children with RMS of the hand or foot treated with local radiotherapy achieved excellent (100%) local control and recommended definitive RT or surgical resection that maintains form and function as the primary local therapy. As an important prognostic factor, the evaluation of lymph node metastasis has become an important basis for decisions regarding postoperative intervention^21^. A previous study^20^ showed that patients with nonmetastatic RMS of the extremity with clinical or pathologic evidence of either in-transit and/or proximal lymph node involvement at diagnosis should undergo appropriate radiotherapy delivered to the in-transit nodal site and/or proximal lymph node site. It is interesting that in patients who have a higher probability of 5-year survival (low-intermediate risk), surgery and radiotherapy greatly improve patient prognosis.

**Table 2:** Multivariable analysis for overall survival in children with rhabdomyosarcoma^#^.

Characteristic		n (%)	Multivariable analysis		
			HR	95% CI	p
Age group	≥10	88 (44.7)	baseline		
	1–9	83 (42.1)	0.528	0.317–0.880	0.014*
	≤1	26 (13.2)	0.362	0.140–0.937	0.036*
Tumor location	Upper limb, shoulder	90 (45.7)	baseline		
	Lower limb, hip	107 (54.3)	0.907	0.546–1.505	0.705
Tumor size	≤5 cm	82 (41.6)	baseline		
	>5 cm	102 (51.8)	1.530	0.859–2.725	0.149
	Unknown	13 (6.6)	1.613	0.616–4.221	0.330
N classification	Negative	90 (45.7)	baseline		
	Positive	65 (33.0)	2.036	1.179–3.518	0.011*
	Unknown	42 (21.3)	1.931	1.057–3.527	0.032
M classification	M0	130 (66.0)	baseline		
	M1	63 (32.0)	3.689	2.118–6.423	<0.001*
	Unknown	4 (2.0)	0.455	0.058–3.581	0.454
Grade	Grade I/II/III	25 (12.7)	baseline		
	Grade IV	31 (15.7)	0.513	0.216–1.218	0.130
	Unknown	141 (71.6)	0.824	0.405–1.679	0.824
Histology	Embryonal	27 (13.7)	baseline		
	Alveolar	138 (70.1)	1.654	0.800–3.420	0.174
	Others	32 (16.2)	1.909	0.769–4.741	0.164
Treatment combinations	Surgery without radiation	42 (21.3)	baseline		
	Surgery + radiation	94 (47.7)	0.471	0.254–0.875	0.017*
	Radiation without surgery	61 (31.0)	0.604	0.325–1.122	0.110

^*^Statistical significance.

^#^We did not include the IRSG staging system, IRSG surgical-pathologic grouping system or IRSG prognostic stratification model in the final Cox proportional hazards multivariable regression model because they are systematic prognostic systems containing several prognostic factors (Supplementary Tables 1 and 2).

Further, we also concluded that the survival advantage in patients who received surgery and radiation disappeared in patients with high risk. According to our RPA (Fig. 3) and IRSG prognostic stratification model (Supplementary Table 0,2), all the high risk cohorts were metastasis patients. This suggests that in high-risk patients, local interventions (surgery and radiotherapy) should be relegated to a secondary position, and anything that would slow down systemic therapy should be minimized in the treatment paradigm. Unfortunately, due to the SEER’s lack of the sequences and timing of local and systemic therapy, we cannot demonstrate more valuable clinical information, and more studies are needed. Taken together, we can justify the use of surgery and radiotherapy in patients who have a higher probability of 5-year survival and do not recommend more aggressive surgery combined with radiotherapy in patients who have a lower probability of 5-year survival (high risk).

Interestingly, in our study, we demonstrated that tumor size was not a significant factor in childhood RMS of the extremities, which is consistent with several previous studies^15,20,21^. The outcome for children with extremity RMS remains suboptimal compared with that of children with RMS in more favorable sites, and the extremity sites are often associated with additional poor prognostic factors, such as alveolar histology and regional nodal involvement^15^. Therefore, the potential influence of tumor size may be diluted by other influential prognostic factors. Thus, in childhood RMS of the extremities, tumor size may not be an important basis for decisions regarding intervention.

As noted previously, our study had several limitations. First, the SEER database does not provide sufficient data regarding the detailed regimens for chemotherapy or radiotherapy. Second, we were unable to conduct independent external validation to confirm the performance of the nomogram. Finally, the retrospective nature of our analysis inevitably produces an inherent bias. Nevertheless, the SEER registry provides critical information regarding the basic characteristics and treatment of children with RMS. The merits of this SEER data outweigh the limitations for the assessment of patient survival.

## Conclusion

We performed a population-based analysis of data from the SEER registry to identify prognostic factors and develop a nomogram in children with extremity RMS. The nomogram appears to be suitable for survival stratification in children with RMS and will help clinicians identify patients who may have a reduced probability of overall survival and assist them in making treatment and surveillance decisions. More studies concerning overall survival in children with RMS are needed to confirm and update our findings.

## Supplementary information

Supplementary information

Supplementary information 2
