# Supplementary material for: Survival stratification in childhood rhabdomyosarcoma of the extremities: a derivation and validation study
Source: Sci Rep. 2020 Mar 30;10:5684. doi: 10.1038/s41598-020-62656-x (PMC7105456; doi:10.1038/s41598-020-62656-x)
Supplement: Supplementary file 1 — Supplementary information [file 41598_2020_62656_MOESM1_ESM.pdf]

Supplementary table 01. IRSG staging system, IRSG surgical-pathologic grouping system and AJCC staging system

**IRSG staging system**				
Stage	Sites of primary tumor	Tumor size	Regional lymph nodes	Distant metastases
1	Orbit, non-parameningeal head/neck; genito-urinary non-bladder/prostate; biliary tract	Any size	N0, N1	M0
2	All other sites	≤5cm	N0	M0
3	All other sites	≤5cm	N1	M0
		>5cm	N0 or N1	
4	Any site	Any size	N0 or N1	M1
**IRSG surgical-pathologic grouping system**				
I	Localized tumor, completely removed with pathologically clear margins and no regional lymph node involvement			
II	Localized tumor, grossly removed with (a) microscopically involved margins, (b) involved, grossly resected regional lymph nodes, or (c) both			
III	Localized tumor, with gross residual disease after grossly incomplete removal, or biopsy only			
IV	Distant metastases present at diagnosis			
**AJCC staging system(8th edition)**				
	Grade	Primary Tumor	Regional Lymph Node	Distant Metastasis
IA	G1, Gx	T1	N0	M0
IB	G1, Gx	T2, T3, T4	N0	M0
II	G2, G3	T1	N0	M0
IIIA	G2, G3	T2	N0	M0
IIIB	G2, G3	T3, T4	N0	M0
IV	Any G	Any T	N1	M0
IV	Any G	Any T	Any N	M1

Definitions:

**IRSG staging system**		
Regional lymph nodes	N0	regional nodes not clinically involved
	N1	regional nodes clinically involved by neoplasm
Distant metastases	M0	no distant metastasis
	M1	metastasis present
**AJCC staging system(8th edition)**		
Grade	Gx	Grade cannot be assessed
	G1	Total differentiation, mitotic count and necrosis score of 2 or 3
	G2	Total differentiation, mitotic count and necrosis score of 4 or 5
	G3	Total differentiation, mitotic count and necrosis score of 6 ,7 , or 8
Primary Tumor	Tx	Primary tumor cannot be assessed
	T0	No evidence of primary tumor
	T1	Tumor 5 cm or less in greatest dimension
	T2	Tumor more than 5 cm and less than or equal to 10 cm in greatest dimension

	T3	Tumor more than 10 cm and less than or equal to 15 cm in greatest dimension
	T4	Tumor more than 15 cm in greatest dimension
Regional Lymph Node	N0	No regional lymph node metastasis or unknown lymph node status
	N1	Regional lymph node metastasis
Distant Metastasis	M0	No distant metastasis
	M1	Distant metastasis

Title: Survival stratification in childhood rhabdomyosarcoma of the extremities: a derivation and validation study

Running title: Survival stratification in childhood rhabdomyosarcoma

Authors: Linchao Zhu, MD[1]; Ying Sun[2]; Xuhui Wang, MD[1]; Lin Wang[1]; Shufeng Zhang[1]; Qinglei Meng[1]; Xiaohui Wang, MD[1]

Author Affiliations:

1 Department of Pediatric Surgery, Henan Provincial People's Hospital, Zhengzhou 450000, Henan Province, China

2 Department of Clinical Laboratory, Third People's Hospital of Henan Province, Zhengzhou 450000, Henan Province, China
