# Supplementary material for: Survival stratification in childhood rhabdomyosarcoma of the extremities: a derivation and validation study
Source: Sci Rep. 2020 Mar 30;10:5684. doi: 10.1038/s41598-020-62656-x (PMC7105456; doi:10.1038/s41598-020-62656-x)
Supplement: Supplementary file 2 — Supplementary information 2 [file 41598_2020_62656_MOESM2_ESM.pdf]

Supplementary table 02. IRSG prognostic stratification

Risk	Stage	Group	Site*	Size$	Age (years)	Histology#	Metastasis±	Nodesɨ	Treatment※
Low, subgroup A	1	I	Favorable	a or b	< 21	EMB	M0	N0	VA
	1	II	Favorable	a or b	< 21	EMB	M0	N0	VA + XRT
	1	III	Orbit only	a or b	< 21	EMB	M0	N0	VA + XRT
	2	I	Unfavorable	a	< 21	EMB	M0	N0 or Nx	VA
Low, subgroup B	1	II	Favorable	a or b	< 21	EMB	M0	N1	VAC + XRT
	1	III	Orbit only	a or b	< 21	EMB	M0	N1	VAC + XRT
	1	III	Favorable (excluding orbit)	a or b	< 21	EMB	M0	N0 or N1 or Nx	VAC + XRT
	2	II	Unfavorable	a	< 21	EMB	M0	N0 or Nx	VAC + XRT
	3	I or II	Unfavorable	a	< 21	EMB	M0	N1	VAC (+ XRT, Gp II)
	3	I or II	Unfavorable	b	< 21	EMB	M0	N0 or N1 or Nx	VAC (+ XRT, Gp II)
Intermediate	2	III	Unfavorable	a	< 21	EMB	M0	N0 or Nx	VAC ±Topo + XRT
	3	III	Unfavorable	a	< 21	EMB	M0	N1	VAC ±Topo + XRT
	3	III	Unfavorable	b	< 21	EMB	M0	N0 or N1 or Nx	VAC ±Topo + XRT
	1 or 2 or 3	I or II or III	Favorable or unfavorable	a or b	< 21	ALV/UDS	M0	N0 or N1 or Nx	VAC ±Topo + XRT
	4	I or II or III or IV	Favorable or unfavorable	a or b	< 10	EMB	M1	N0 or N1 or Nx	VAC ±Topo + XRT
High	4	IV	Favorable or unfavorable	a or b	≥10	EMB	M1	N0 or N1 or Nx	CPT-11, VAC + XRT
	4	IV	Favorable or unfavorable	a or b	< 21	ALV/UDS	M1	N0 or N1 or Nx	CPT-11, VAC + XRT

Denefitions:

* Favorable, orbit/eyelid, head and neck (excluding parameningeal), genito-urinary (not bladder or prostate); biliary tract; unfavorable, bladder, prostate, extremity, parameningeal, trunk, retroperitoneal, pelvis, other.

$ a, tumor size ≤ 5 cm in diameter; b, tumor size > 5 cm in diameter.

EMB, embryonal, botryoid or spindle-cell rhabdomyosarcomas or ectomesenchymonas with embryonal RMS; ALV, alveolar rhabdomyosarcomas, or ectomesenchymomas with alveolar RMS; UDS, undifferentiated sarcomas.

± M0, no distant metastases; M1, distant metastases at diagnosis.

ǂ N0, regional nodes clinically not involved; N1, regional nodes clinically involved; NX, node status unknown.

※ VAC, vincristine, actinomycin D, cyclophosphamide; XRT, radiotherapy; Topo, topotecan; Gp, group; CPT-11, irinotecan.

Title: Survival stratification in childhood rhabdomyosarcoma of the extremities: a derivation and validation study

Running title: Survival stratification in childhood rhabdomyosarcoma

Authors: Linchao Zhu, MD[1]; Ying Sun[2]; Xuhui Wang, MD[1]; Lin Wang[1]; Shufeng Zhang[1]; Qinglei Meng[1]; Xiaohui Wang, MD[1]

Author Affiliations:

1 Department of Pediatric Surgery, Henan Provincial People's Hospital, Zhengzhou 450000, Henan Province, China

2 Department of Clinical Laboratory, Third People's Hospital of Henan Province, Zhengzhou 450000, Henan Province, China
